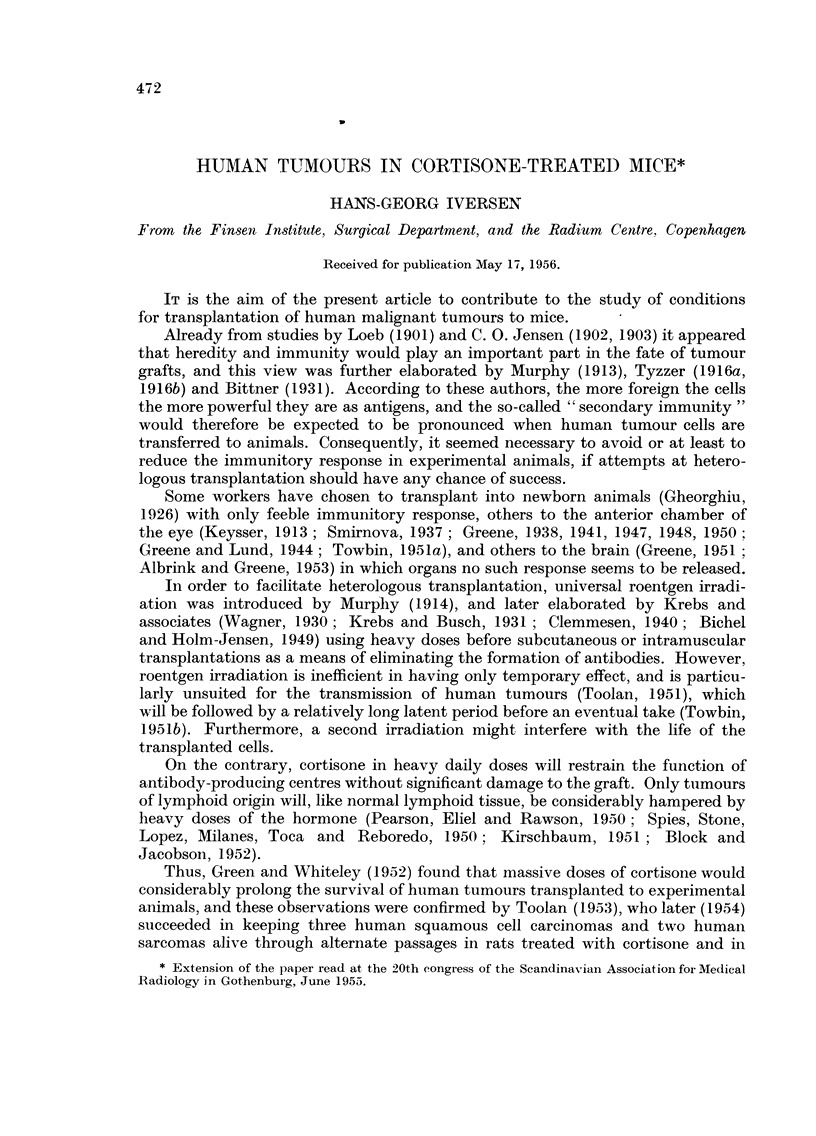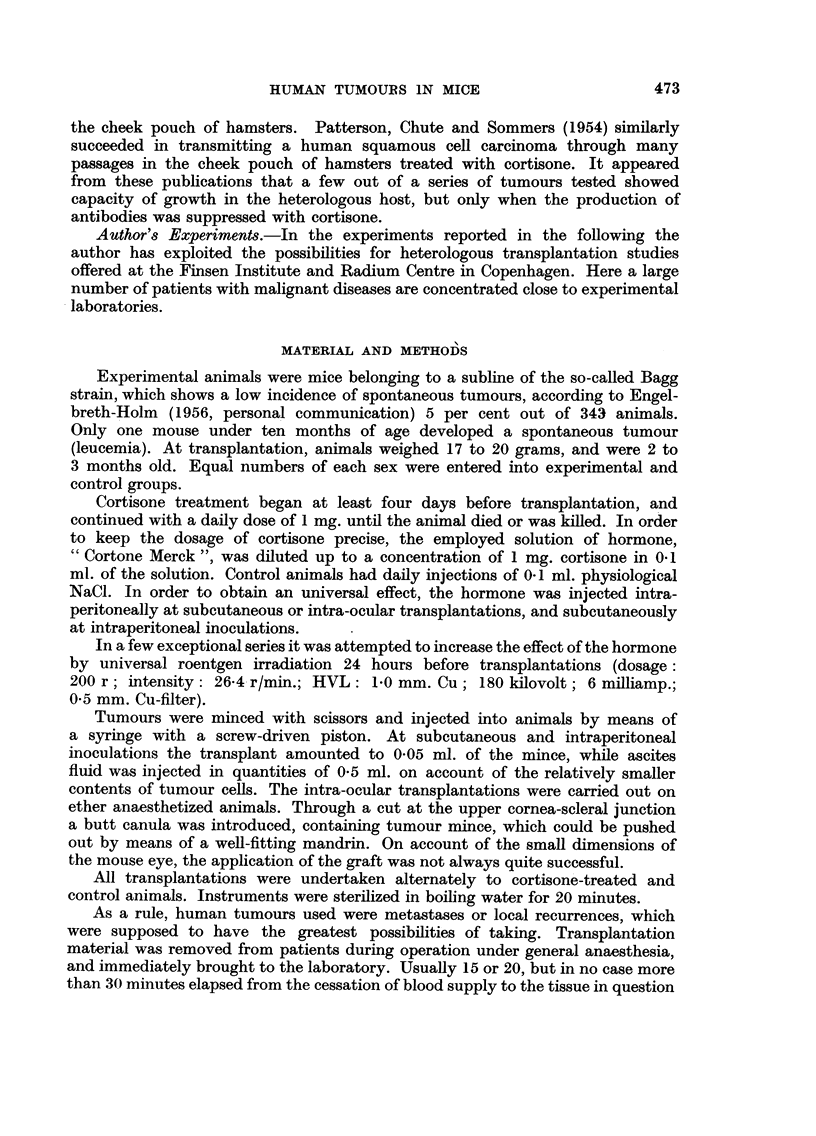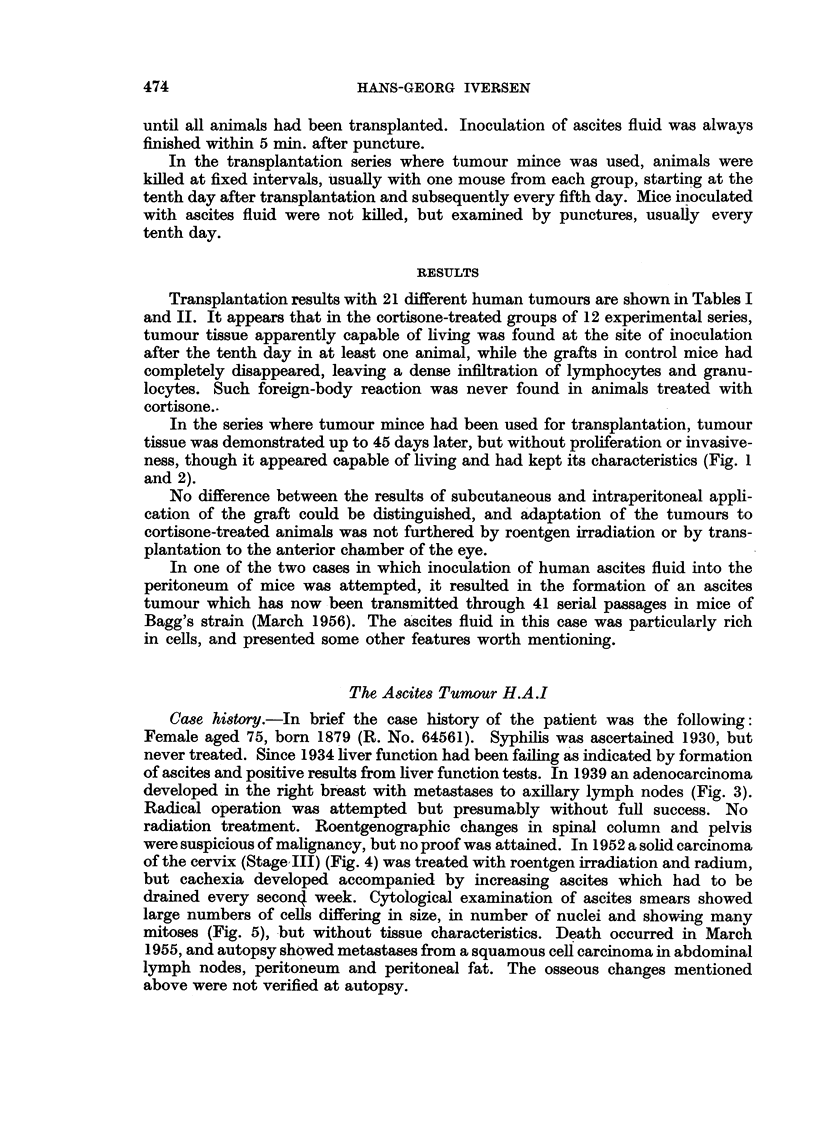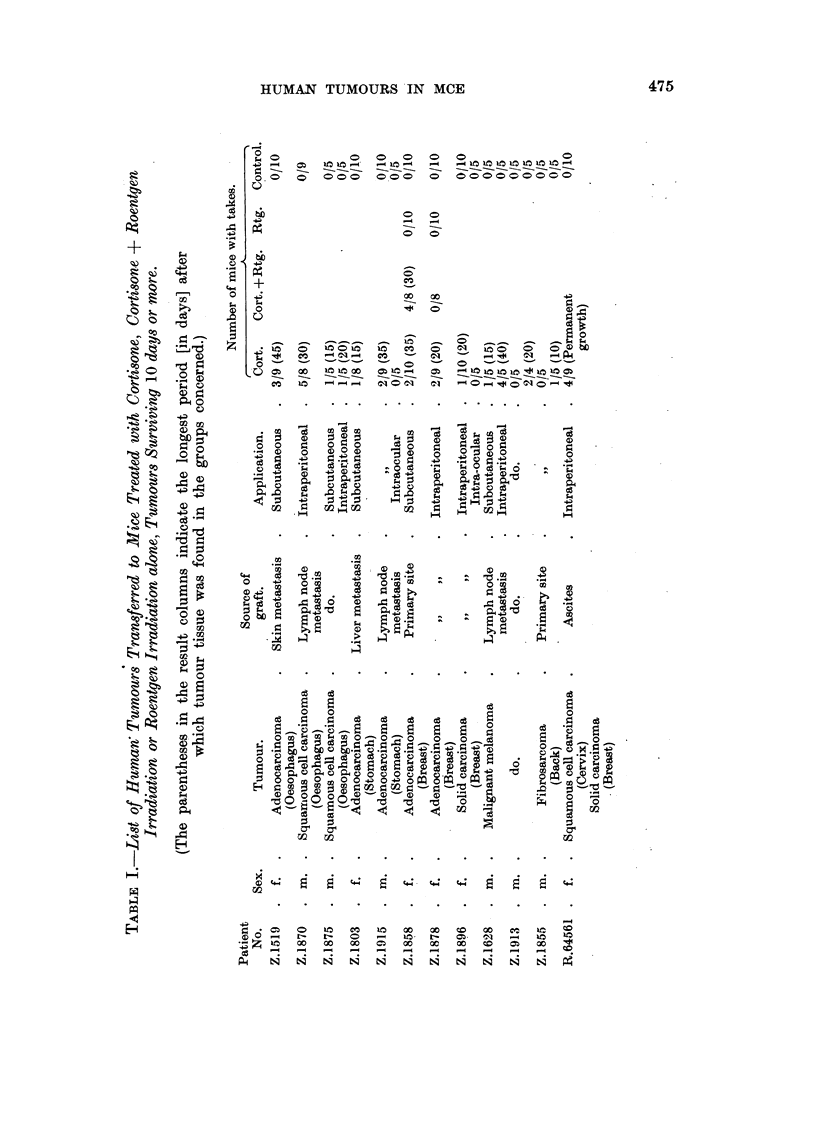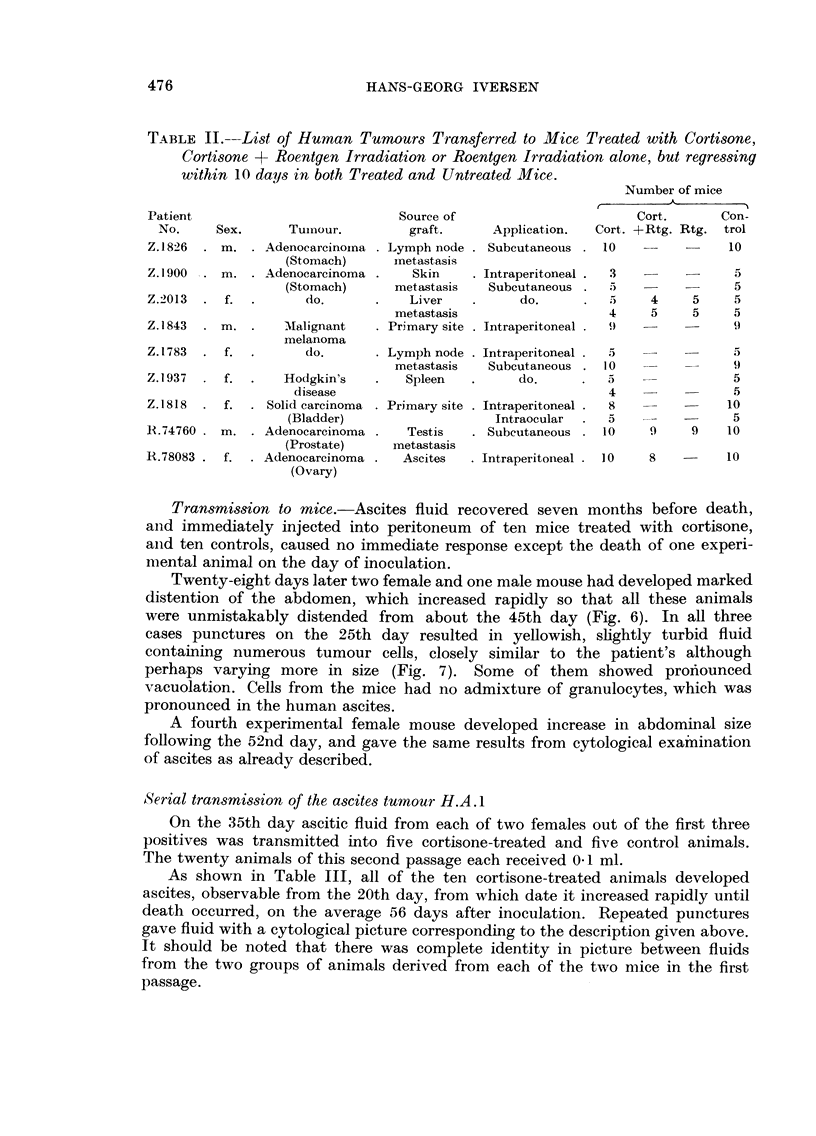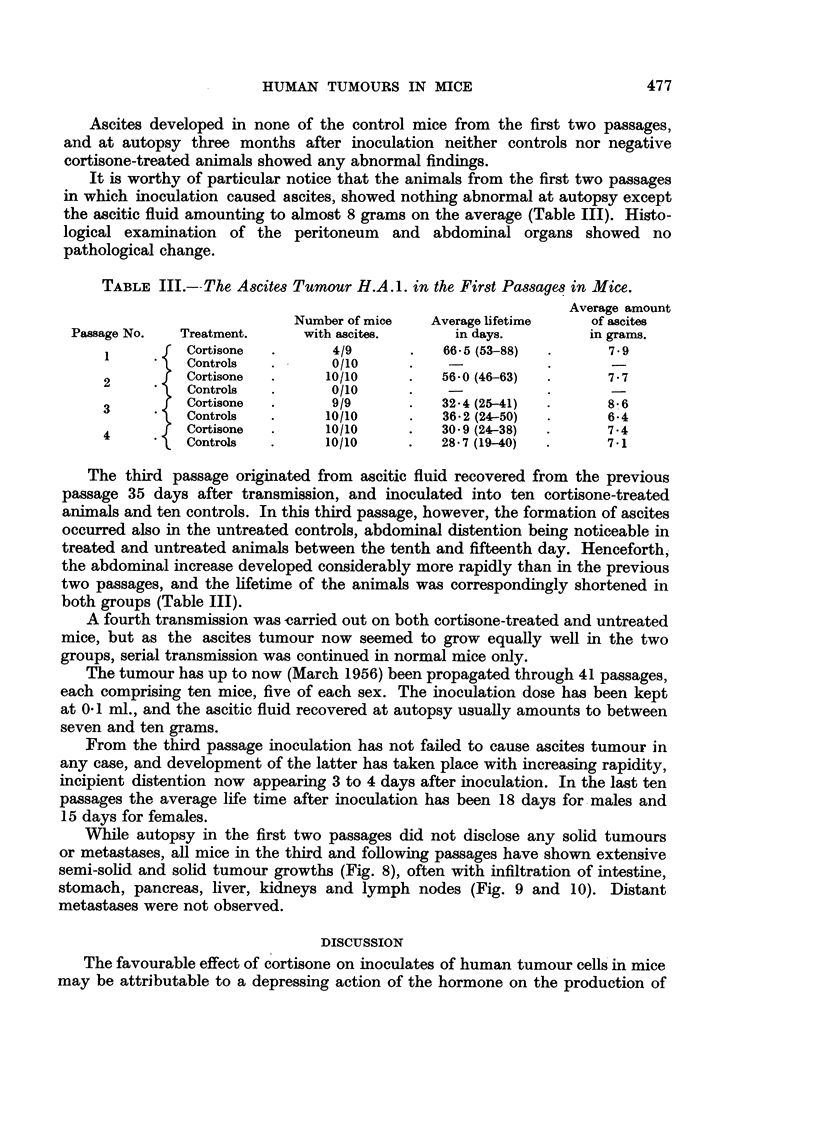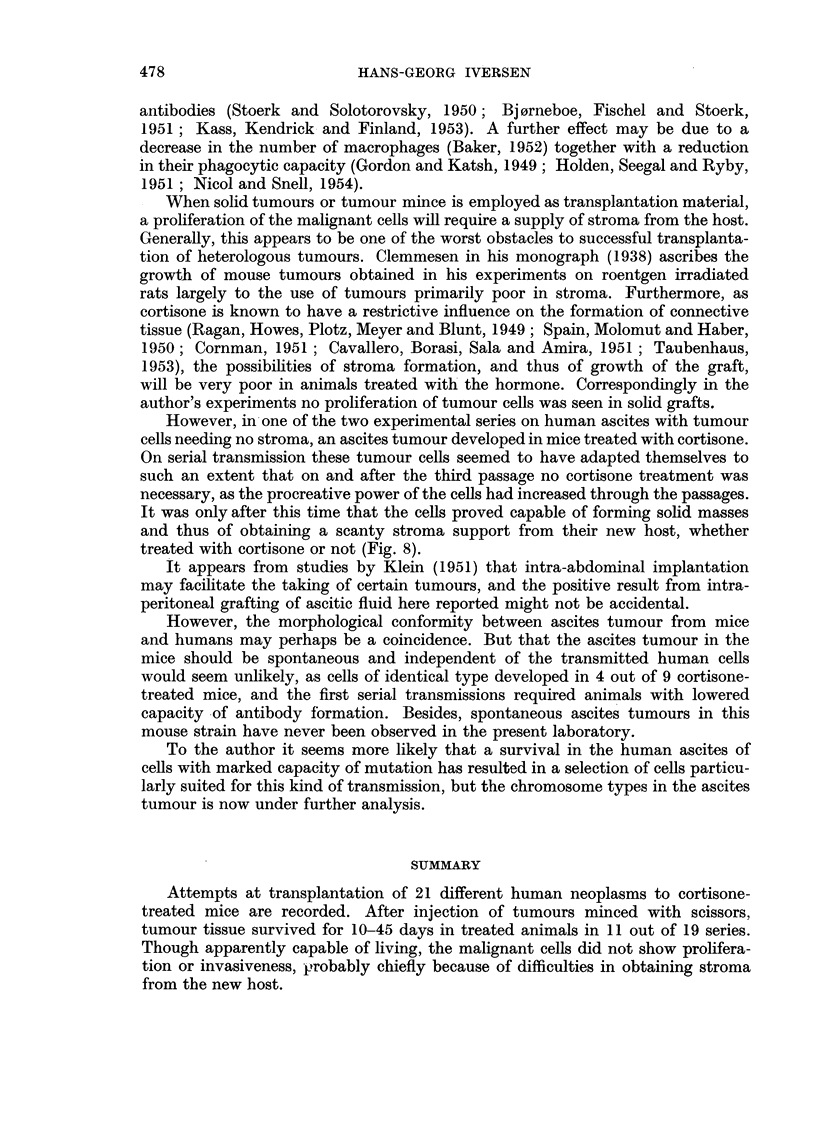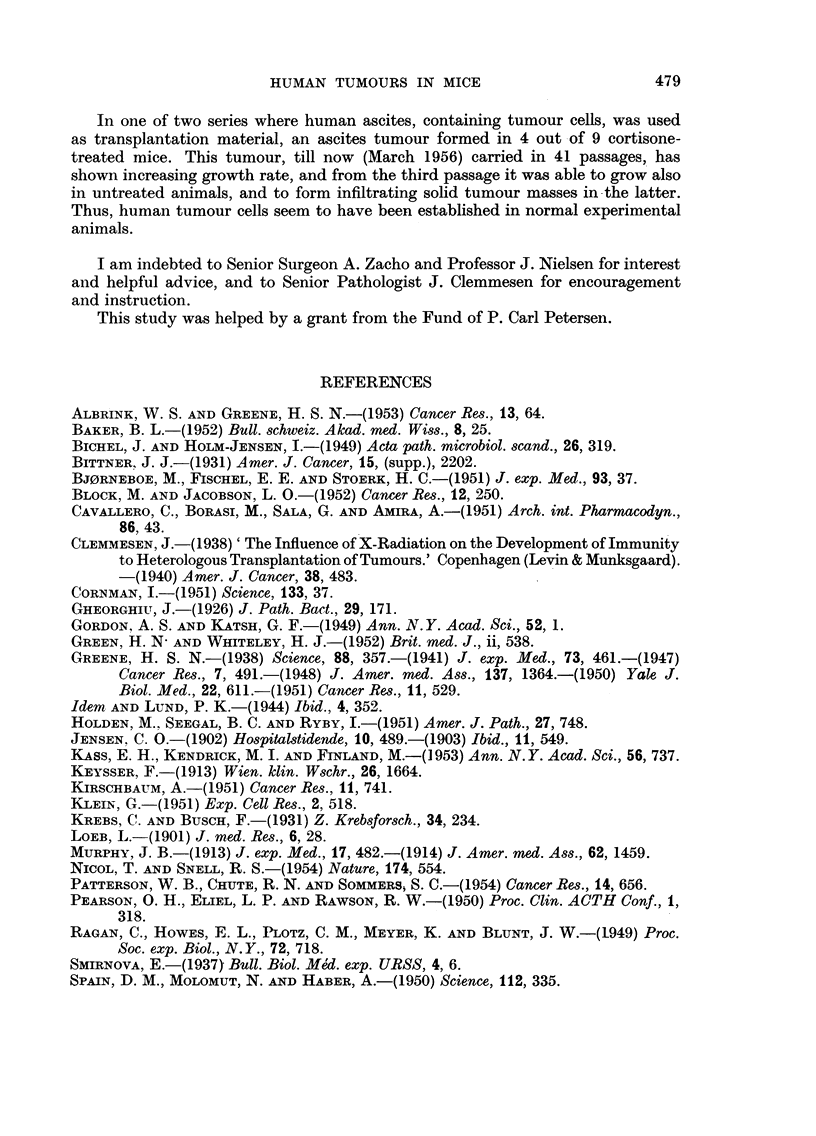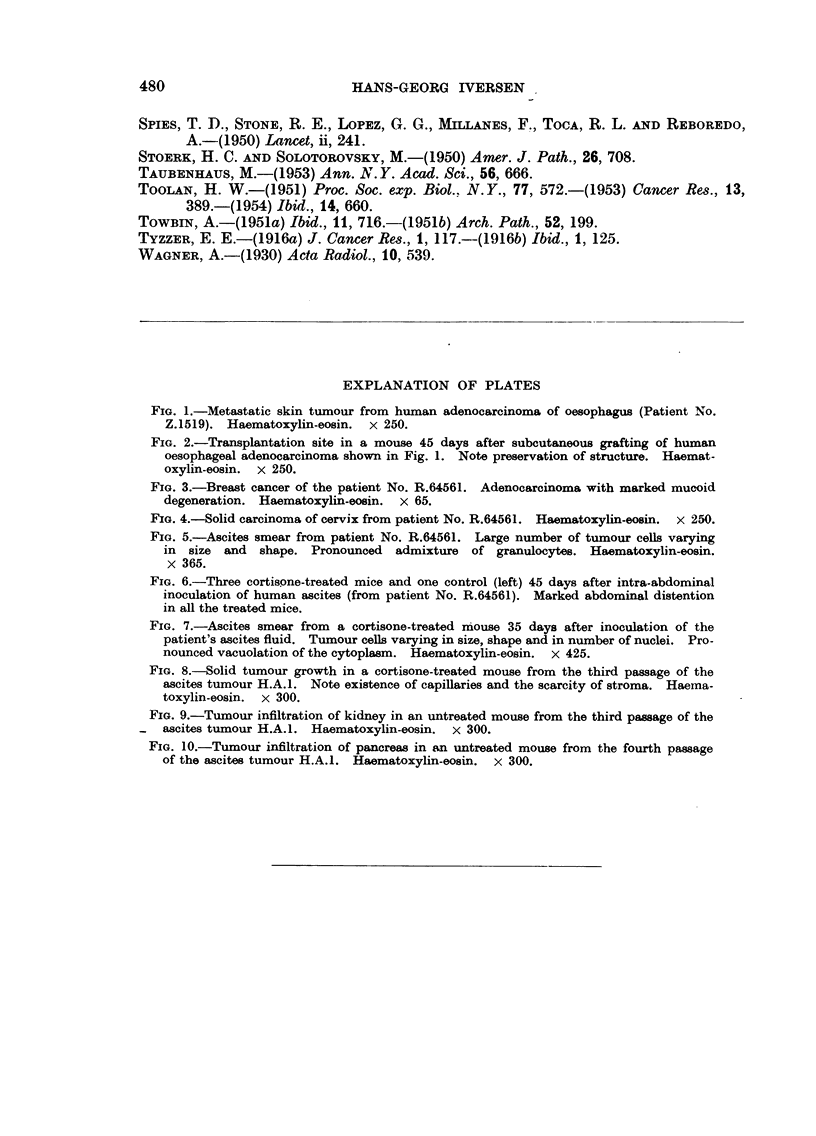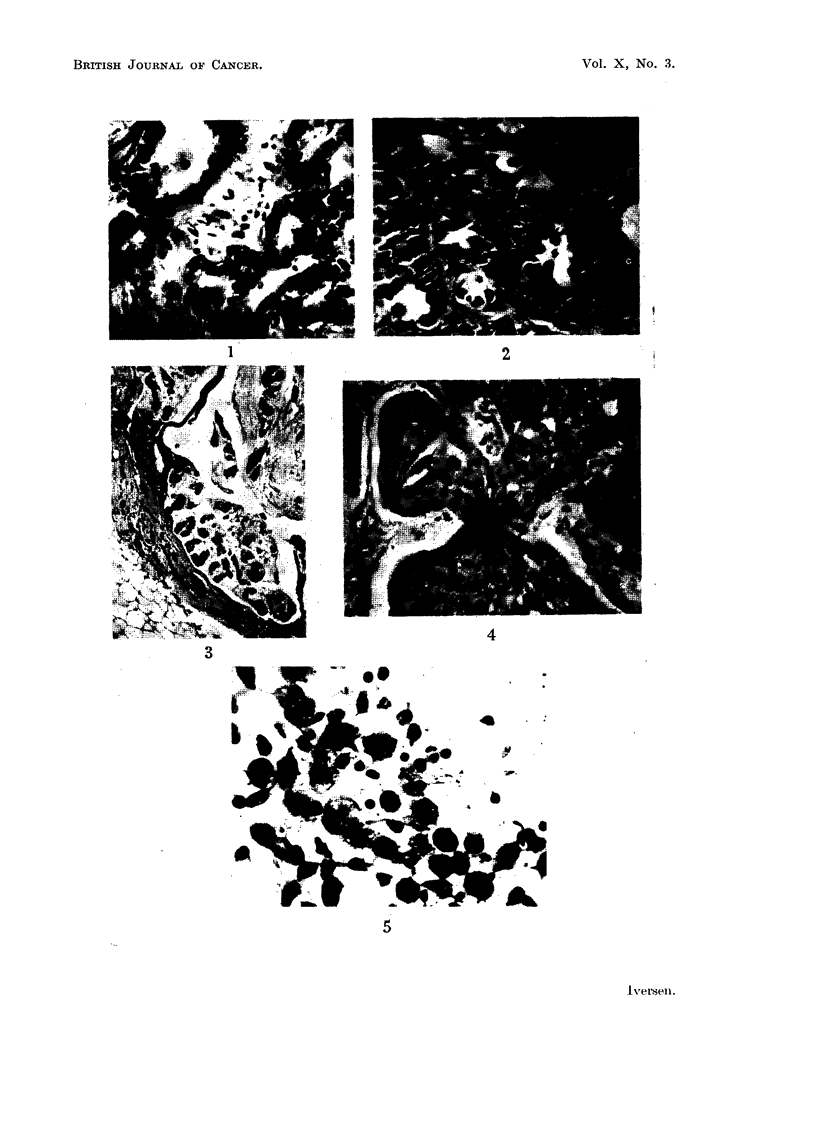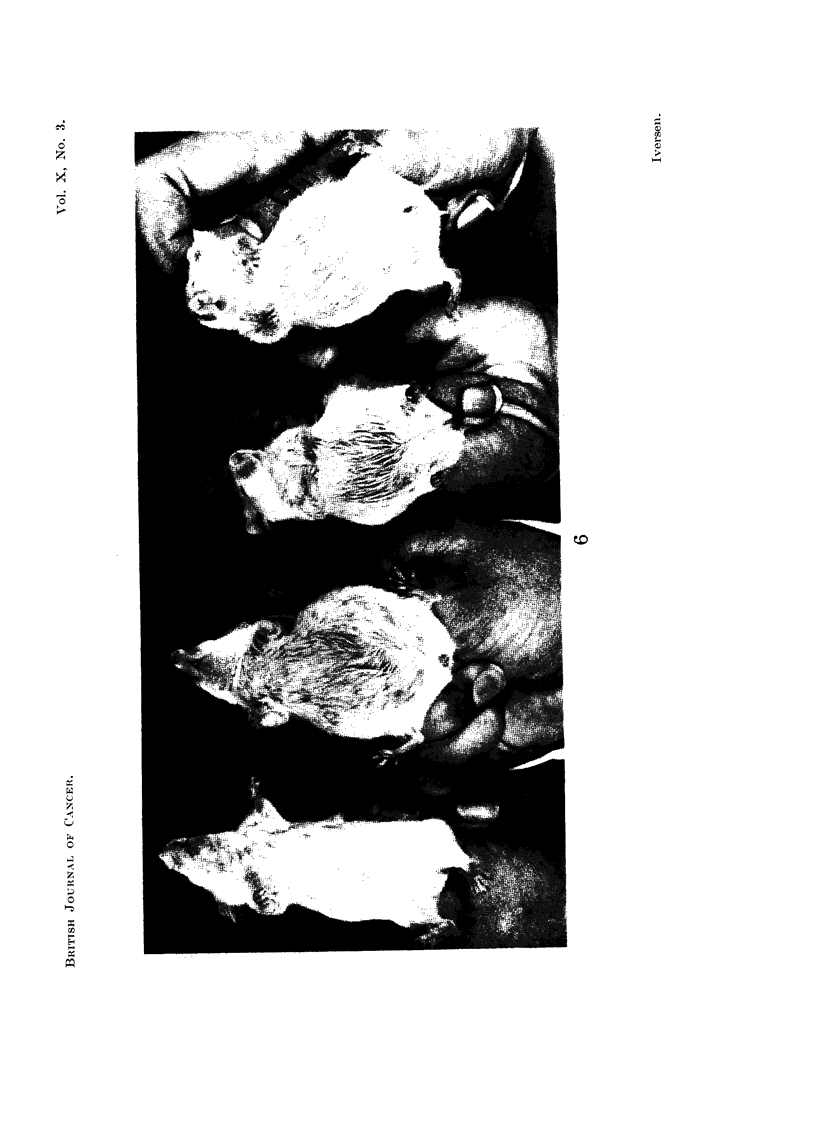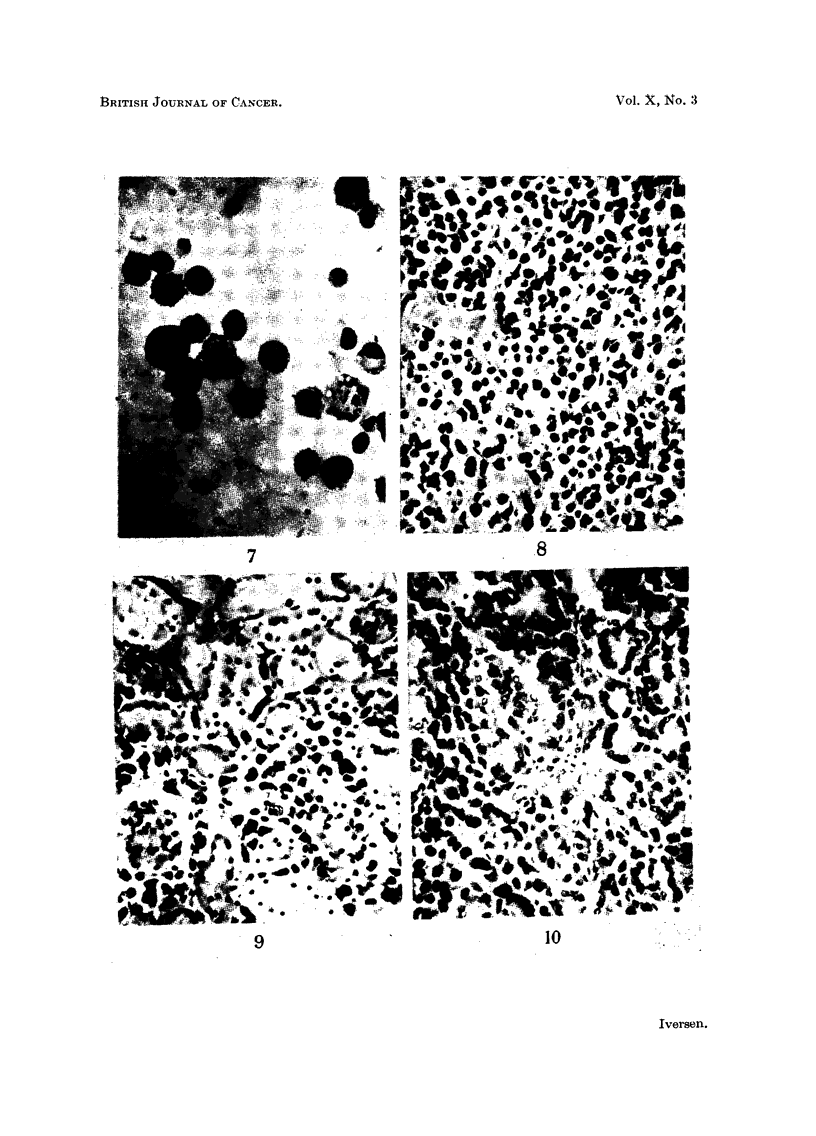# Human Tumours in Cortisone-Treated Mice*

**DOI:** 10.1038/bjc.1956.53

**Published:** 1956-09

**Authors:** Hans-Georg Iversen

## Abstract

**Images:**


### HUMAN TUMOURS IN CORTISONE-TREATED MICE*

HANS-GEORG IVERSEN

*From the Finsen Institute, Surgical Department, and the Radium Centre, Copenhagen*

Received for publication May 17, 1956.

IT is the aim of the present article to contribute to the study of conditions for transplantation of human malignant tumours to mice.

Already from studies by Loeb (1901) and C. O. Jensen (1902, 1903) it appeared that heredity and immunity would play an important part in the fate of tumour grafts, and this view was further elaborated by Murphy (1913), Tyzzer (1916*a*, 1916*b*) and Bittner (1931). According to these authors, the more foreign the cells the more powerful they are as antigens, and the so-called "secondary immunity" would therefore be expected to be pronounced when human tumour cells are transferred to animals. Consequently, it seemed necessary to avoid or at least to reduce the immunitory response in experimental animals, if attempts at heterologous transplantation should have any chance of success.

Some workers have chosen to transplant into newborn animals (Gheorghiu, 1926) with only feeble immunitory response, others to the anterior chamber of the eye (Keysser, 1913 ; Smirnova, 1937 ; Greene, 1938, 1941, 1947, 1948, 1950 ; Greene and Lund, 1944 ; Towbin, 1951*a*), and others to the brain (Greene, 1951 ; Albrink and Greene, 1953) in which organs no such response seems to be released.

In order to facilitate heterologous transplantation, universal roentgen irradiation was introduced by Murphy (1914), and later elaborated by Krebs and associates (Wagner, 1930 ; Krebs and Busch, 1931 ; Clemmesen, 1940 ; Bichel and Holm-Jensen, 1949) using heavy doses before subcutaneous or intramuscular transplantations as a means of eliminating the formation of antibodies. However, roentgen irradiation is inefficient in having only temporary effect, and is particularly unsuited for the transmission of human tumours (Toolan, 1951), which will be followed by a relatively long latent period before an eventual take (Towbin, 1951*b*). Furthermore, a second irradiation might interfere with the life of the transplanted cells.

On the contrary, cortisone in heavy daily doses will restrain the function of antibody-producing centres without significant damage to the graft. Only tumours of lymphoid origin will, like normal lymphoid tissue, be considerably hampered by heavy doses of the hormone (Pearson, Eliel and Rawson, 1950 ; Spies, Stone, Lopez, Milanes, Toca and Reboredo, 1950 ; Kirschbaum, 1951 ; Block and Jacobson, 1952).

Thus, Green and Whiteley (1952) found that massive doses of cortisone would considerably prolong the survival of human tumours transplanted to experimental animals, and these observations were confirmed by Toolan (1953), who later (1954) succeeded in keeping three human squamous cell carcinomas and two human sarcomas alive through alternate passages in rats treated with cortisone and in

* Extension of the paper read at the 20th congress of the Scandinavian Association for Medical Radiology in Gothenburg, June 1955.

the cheek pouch of hamsters. Patterson, Chute and Sommers (1954) similarly succeeded in transmitting a human squamous cell carcinoma through many passages in the cheek pouch of hamsters treated with cortisone. It appeared from these publications that a few out of a series of tumours tested showed capacity of growth in the heterologous host, but only when the production of antibodies was suppressed with cortisone.

*Author's Experiments.*—In the experiments reported in the following the author has exploited the possibilities for heterologous transplantation studies offered at the Finsen Institute and Radium Centre in Copenhagen. Here a large number of patients with malignant diseases are concentrated close to experimental laboratories.

### MATERIAL AND METHODS

Experimental animals were mice belonging to a subline of the so-called Bagg strain, which shows a low incidence of spontaneous tumours, according to Engelbreth-Holm (1956, personal communication) 5 per cent out of 343 animals. Only one mouse under ten months of age developed a spontaneous tumour (leucemia). At transplantation, animals weighed 17 to 20 grams, and were 2 to 3 months old. Equal numbers of each sex were entered into experimental and control groups.

Cortisone treatment began at least four days before transplantation, and continued with a daily dose of 1 mg. until the animal died or was killed. In order to keep the dosage of cortisone precise, the employed solution of hormone, "Cortone Merck", was diluted up to a concentration of 1 mg. cortisone in 0·1 ml. of the solution. Control animals had daily injections of 0·1 ml. physiological NaCl. In order to obtain an universal effect, the hormone was injected intraperitoneally at subcutaneous or intra-ocular transplantations, and subcutaneously at intraperitoneal inoculations.

In a few exceptional series it was attempted to increase the effect of the hormone by universal roentgen irradiation 24 hours before transplantations (dosage: 200 r; intensity: 26·4 r/min.; HVL: 1·0 mm. Cu; 180 kilovolt; 6 milliamp.; 0·5 mm. Cu-filter).

Tumours were minced with scissors and injected into animals by means of a syringe with a screw-driven piston. At subcutaneous and intraperitoneal inoculations the transplant amounted to 0·05 ml. of the mince, while ascites fluid was injected in quantities of 0·5 ml. on account of the relatively smaller contents of tumour cells. The intra-ocular transplantations were carried out on ether anaesthetized animals. Through a cut at the upper cornea-scleral junction a butt canula was introduced, containing tumour mince, which could be pushed out by means of a well-fitting mandrin. On account of the small dimensions of the mouse eye, the application of the graft was not always quite successful.

All transplantations were undertaken alternately to cortisone-treated and control animals. Instruments were sterilized in boiling water for 20 minutes.

As a rule, human tumours used were metastases or local recurrences, which were supposed to have the greatest possibilities of taking. Transplantation material was removed from patients during operation under general anaesthesia, and immediately brought to the laboratory. Usually 15 or 20, but in no case more than 30 minutes elapsed from the cessation of blood supply to the tissue in question until all animals had been transplanted. Inoculation of ascites fluid was always finished within 5 min. after puncture.

In the transplantation series where tumour mince was used, animals were killed at fixed intervals, usually with one mouse from each group, starting at the tenth day after transplantation and subsequently every fifth day. Mice inoculated with ascites fluid were not killed, but examined by punctures, usually every tenth day.

### RESULTS

Transplantation results with 21 different human tumours are shown in Tables I and II. It appears that in the cortisone-treated groups of 12 experimental series, tumour tissue apparently capable of living was found at the site of inoculation after the tenth day in at least one animal, while the grafts in control mice had completely disappeared, leaving a dense infiltration of lymphocytes and granulocytes. Such foreign-body reaction was never found in animals treated with cortisone..

In the series where tumour mince had been used for transplantation, tumour tissue was demonstrated up to 45 days later, but without proliferation or invasiveness, though it appeared capable of living and had kept its characteristics (Fig. 1 and 2).

No difference between the results of subcutaneous and intraperitoneal application of the graft could be distinguished, and adaptation of the tumours to cortisone-treated animals was not furthered by roentgen irradiation or by transplantation to the anterior chamber of the eye.

In one of the two cases in which inoculation of human ascites fluid into the peritoneum of mice was attempted, it resulted in the formation of an ascites tumour which has now been transmitted through 41 serial passages in mice of Bagg's strain (March 1956). The ascites fluid in this case was particularly rich in cells, and presented some other features worth mentioning.

### *The Ascites Tumour H.A.I*

*Case history.*—In brief the case history of the patient was the following: Female aged 75, born 1879 (R. No. 64561). Syphilis was ascertained 1930, but never treated. Since 1934 liver function had been failing as indicated by formation of ascites and positive results from liver function tests. In 1939 an adenocarcinoma developed in the right breast with metastases to axillary lymph nodes (Fig. 3). Radical operation was attempted but presumably without full success. No radiation treatment. Roentgenographic changes in spinal column and pelvis were suspicious of malignancy, but no proof was attained. In 1952 a solid carcinoma of the cervix (Stage III) (Fig. 4) was treated with roentgen irradiation and radium, but cachexia developed accompanied by increasing ascites which had to be drained every second week. Cytological examination of ascites smears showed large numbers of cells differing in size, in number of nuclei and showing many mitoses (Fig. 5), but without tissue characteristics. Death occurred in March 1955, and autopsy showed metastases from a squamous cell carcinoma in abdominal lymph nodes, peritoneum and peritoneal fat. The osseous changes mentioned above were not verified at autopsy.

TABLE I.—*List of Human Tumours Transferred to Mice Treated with Cortisone, Cortisone + Roentgen Irradiation or Roentgen Irradiation alone, Tumours Surviving* 10 *days or more.*

(The parentheses in the result columns indicate the longest period [in days] after which tumour tissue was found in the groups concerned.)

| Patient No. | Sex. | Tumour. | Source of graft. | Application. | Number of mice with takes. Cort. | Cort. + Rtg. | Rtg. | Control. |
|---|---|---|---|---|---|---|---|---|
| Z.1519 | f. | Adenocarcinoma (Oesophagus) | Skin metastasis | Subcutaneous | 3/9 (45) | | | 0/10 |
| Z.1870 | m. | Squamous cell carcinoma (Oesophagus) | Lymph node metastasis | Intraperitoneal | 5/8 (30) | | | 0/9 |
| Z.1875 | m. | Squamous cell carcinoma (Oesophagus) | do. | Subcutaneous | 1/5 (15) | | | 0/5 |
| | | | | Intraperitoneal | 1/5 (20) | | | 0/5 |
| Z.1803 | f. | Adenocarcinoma (Stomach) | Liver metastasis | Subcutaneous | 1/8 (15) | | | 0/10 |
| Z.1915 | m. | Adenocarcinoma (Stomach) | Lymph node metastasis | ,, | 2/9 (35) | | | 0/10 |
| | | | | Intraocular | 0/5 | | | 0/5 |
| Z.1858 | f. | Adenocarcinoma (Breast) | Primary site | Subcutaneous | 2/10 (35) | 4/8 (30) | 0/10 | 0/10 |
| Z.1878 | f. | Adenocarcinoma (Breast) | ,, ,, | Intraperitoneal | 2/9 (20) | 0/8 | 0/10 | 0/10 |
| Z.1896 | f. | Solid carcinoma (Breast) | ,, ,, | Intraperitoneal | 1/10 (20) | | | 0/10 |
| | | | | Intra-ocular | 0/5 | | | 0/5 |
| Z.1628 | m. | Malignant melanoma | Lymph node metastasis | Subcutaneous | 1/5 (15) | | | 0/5 |
| | | | | Intraperitoneal | 4/5 (40) | | | 0/5 |
| Z.1913 | m. | do. | do. | do. | 0/5 | | | 0/5 |
| | | | | | 2/4 (20) | | | 0/5 |
| Z.1855 | m. | Fibrosarcoma (Back) | Primary site | ,, | 0/5 | | | 0/5 |
| | | | | | 1/5 (10) | | | 0/5 |
| R.64561 | f. | Squamous cell carcinoma (Cervix) Solid carcinoma (Breast) | Ascites | Intraperitoneal | 4/9 (Permanent growth) | | | 0/10 |

TABLE II.—*List of Human Tumours Transferred to Mice Treated with Cortisone, Cortisone + Roentgen Irradiation or Roentgen Irradiation alone, but regressing within 10 days in both Treated and Untreated Mice.*

| Patient No. | Sex. | Tumour. | Source of graft. | Application. | Number of mice | | | |
|---|---|---|---|---|---|---|---|---|
| | | | | | Cort. | Cort. +Rtg. | Rtg. | Control |
| Z.1826 | m. | Adenocarcinoma (Stomach) | Lymph node metastasis | Subcutaneous | 10 | — | — | 10 |
| Z.1900 | m. | Adenocarcinoma (Stomach) | Skin metastasis | Intraperitoneal | 3 | — | — | 5 |
| | | | | Subcutaneous | 5 | — | — | 5 |
| Z.2013 | f. | do. | Liver metastasis | do. | 5 | 4 | 5 | 5 |
| | | | | | 4 | 5 | 5 | 5 |
| Z.1843 | m. | Malignant melanoma | Primary site | Intraperitoneal | 9 | — | — | 9 |
| Z.1783 | f. | do. | Lymph node metastasis | Intraperitoneal | 5 | — | — | 5 |
| | | | | Subcutaneous | 10 | — | — | 9 |
| Z.1937 | f. | Hodgkin's disease | Spleen | do. | 5 | — | | 5 |
| | | | | | 4 | — | — | 5 |
| Z.1818 | f. | Solid carcinoma (Bladder) | Primary site | Intraperitoneal | 8 | — | — | 10 |
| | | | | Intraocular | 5 | — | — | 5 |
| R.74760 | m. | Adenocarcinoma (Prostate) | Testis metastasis | Subcutaneous | 10 | 9 | 9 | 10 |
| R.78083 | f. | Adenocarcinoma (Ovary) | Ascites | Intraperitoneal | 10 | 8 | — | 10 |

*Transmission to mice.*—Ascites fluid recovered seven months before death, and immediately injected into peritoneum of ten mice treated with cortisone, and ten controls, caused no immediate response except the death of one experimental animal on the day of inoculation.

Twenty-eight days later two female and one male mouse had developed marked distention of the abdomen, which increased rapidly so that all these animals were unmistakably distended from about the 45th day (Fig. 6). In all three cases punctures on the 25th day resulted in yellowish, slightly turbid fluid containing numerous tumour cells, closely similar to the patient's although perhaps varying more in size (Fig. 7). Some of them showed pronounced vacuolation. Cells from the mice had no admixture of granulocytes, which was pronounced in the human ascites.

A fourth experimental female mouse developed increase in abdominal size following the 52nd day, and gave the same results from cytological examination of ascites as already described.

*Serial transmission of the ascites tumour H.A.*1

On the 35th day ascitic fluid from each of two females out of the first three positives was transmitted into five cortisone-treated and five control animals. The twenty animals of this second passage each received 0·1 ml.

As shown in Table III, all of the ten cortisone-treated animals developed ascites, observable from the 20th day, from which date it increased rapidly until death occurred, on the average 56 days after inoculation. Repeated punctures gave fluid with a cytological picture corresponding to the description given above. It should be noted that there was complete identity in picture between fluids from the two groups of animals derived from each of the two mice in the first passage.

Ascites developed in none of the control mice from the first two passages, and at autopsy three months after inoculation neither controls nor negative cortisone-treated animals showed any abnormal findings.

It is worthy of particular notice that the animals from the first two passages in which inoculation caused ascites, showed nothing abnormal at autopsy except the ascitic fluid amounting to almost 8 grams on the average (Table III). Histological examination of the peritoneum and abdominal organs showed no pathological change.

TABLE III.—*The Ascites Tumour H.A.*1. *in the First Passages in Mice.*

| Passage No. | Treatment. | Number of mice with ascites. | Average lifetime in days. | Average amount of ascites in grams. |
|---|---|---|---|---|
| 1 | Cortisone | 4/9 | 66·5 (53–88) | 7·9 |
| | Controls | 0/10 | — | — |
| 2 | Cortisone | 10/10 | 56·0 (46–63) | 7·7 |
| | Controls | 0/10 | — | — |
| 3 | Cortisone | 9/9 | 32·4 (25–41) | 8·6 |
| | Controls | 10/10 | 36·2 (24–50) | 6·4 |
| 4 | Cortisone | 10/10 | 30·9 (24–38) | 7·4 |
| | Controls | 10/10 | 28·7 (19–40) | 7·1 |

The third passage originated from ascitic fluid recovered from the previous passage 35 days after transmission, and inoculated into ten cortisone-treated animals and ten controls. In this third passage, however, the formation of ascites occurred also in the untreated controls, abdominal distention being noticeable in treated and untreated animals between the tenth and fifteenth day. Henceforth, the abdominal increase developed considerably more rapidly than in the previous two passages, and the lifetime of the animals was correspondingly shortened in both groups (Table III).

A fourth transmission was carried out on both cortisone-treated and untreated mice, but as the ascites tumour now seemed to grow equally well in the two groups, serial transmission was continued in normal mice only.

The tumour has up to now (March 1956) been propagated through 41 passages, each comprising ten mice, five of each sex. The inoculation dose has been kept at 0·1 ml., and the ascitic fluid recovered at autopsy usually amounts to between seven and ten grams.

From the third passage inoculation has not failed to cause ascites tumour in any case, and development of the latter has taken place with increasing rapidity, incipient distention now appearing 3 to 4 days after inoculation. In the last ten passages the average life time after inoculation has been 18 days for males and 15 days for females.

While autopsy in the first two passages did not disclose any solid tumours or metastases, all mice in the third and following passages have shown extensive semi-solid and solid tumour growths (Fig. 8), often with infiltration of intestine, stomach, pancreas, liver, kidneys and lymph nodes (Fig. 9 and 10). Distant metastases were not observed.

### DISCUSSION

The favourable effect of cortisone on inoculates of human tumour cells in mice may be attributable to a depressing action of the hormone on the production of antibodies (Stoerk and Solotorovsky, 1950 ; Bjørneboe, Fischel and Stoerk, 1951 ; Kass, Kendrick and Finland, 1953). A further effect may be due to a decrease in the number of macrophages (Baker, 1952) together with a reduction in their phagocytic capacity (Gordon and Katsh, 1949 ; Holden, Seegal and Ryby, 1951 ; Nicol and Snell, 1954).

When solid tumours or tumour mince is employed as transplantation material, a proliferation of the malignant cells will require a supply of stroma from the host. Generally, this appears to be one of the worst obstacles to successful transplantation of heterologous tumours. Clemmesen in his monograph (1938) ascribes the growth of mouse tumours obtained in his experiments on roentgen irradiated rats largely to the use of tumours primarily poor in stroma. Furthermore, as cortisone is known to have a restrictive influence on the formation of connective tissue (Ragan, Howes, Plotz, Meyer and Blunt, 1949 ; Spain, Molomut and Haber, 1950 ; Cornman, 1951 ; Cavallero, Borasi, Sala and Amira, 1951 ; Taubenhaus, 1953), the possibilities of stroma formation, and thus of growth of the graft, will be very poor in animals treated with the hormone. Correspondingly in the author's experiments no proliferation of tumour cells was seen in solid grafts.

However, in one of the two experimental series on human ascites with tumour cells needing no stroma, an ascites tumour developed in mice treated with cortisone. On serial transmission these tumour cells seemed to have adapted themselves to such an extent that on and after the third passage no cortisone treatment was necessary, as the procreative power of the cells had increased through the passages. It was only after this time that the cells proved capable of forming solid masses and thus of obtaining a scanty stroma support from their new host, whether treated with cortisone or not (Fig. 8).

It appears from studies by Klein (1951) that intra-abdominal implantation may facilitate the taking of certain tumours, and the positive result from intraperitoneal grafting of ascitic fluid here reported might not be accidental.

However, the morphological conformity between ascites tumour from mice and humans may perhaps be a coincidence. But that the ascites tumour in the mice should be spontaneous and independent of the transmitted human cells would seem unlikely, as cells of identical type developed in 4 out of 9 cortisone-treated mice, and the first serial transmissions required animals with lowered capacity of antibody formation. Besides, spontaneous ascites tumours in this mouse strain have never been observed in the present laboratory.

To the author it seems more likely that a survival in the human ascites of cells with marked capacity of mutation has resulted in a selection of cells particularly suited for this kind of transmission, but the chromosome types in the ascites tumour is now under further analysis.

### SUMMARY

Attempts at transplantation of 21 different human neoplasms to cortisone-treated mice are recorded. After injection of tumours minced with scissors, tumour tissue survived for 10–45 days in treated animals in 11 out of 19 series. Though apparently capable of living, the malignant cells did not show proliferation or invasiveness, probably chiefly because of difficulties in obtaining stroma from the new host.

In one of two series where human ascites, containing tumour cells, was used as transplantation material, an ascites tumour formed in 4 out of 9 cortisone-treated mice. This tumour, till now (March 1956) carried in 41 passages, has shown increasing growth rate, and from the third passage it was able to grow also in untreated animals, and to form infiltrating solid tumour masses in the latter. Thus, human tumour cells seem to have been established in normal experimental animals.

I am indebted to Senior Surgeon A. Zacho and Professor J. Nielsen for interest and helpful advice, and to Senior Pathologist J. Clemmesen for encouragement and instruction.

This study was helped by a grant from the Fund of P. Carl Petersen.

### REFERENCES

Albrink, W. S. and Greene, H. S. N.—(1953) *Cancer Res.*, **13**, 64.
Baker, B. L.—(1952) *Bull. schweiz. Akad. med. Wiss.*, **8**, 25.
Bichel, J. and Holm-Jensen, I.—(1949) *Acta path. microbiol. scand.*, **26**, 319.
Bittner, J. J.—(1931) *Amer. J. Cancer*, **15**, (supp.), 2202.
Bjørneboe, M., Fischel, E. E. and Stoerk, H. C.—(1951) *J. exp. Med.*, **93**, 37.
Block, M. and Jacobson, L. O.—(1952) *Cancer Res.*, **12**, 250.
Cavallero, C., Borasi, M., Sala, G. and Amira, A.—(1951) *Arch. int. Pharmacodyn.*, **86**, 43.
Clemmesen, J.—(1938) 'The Influence of X-Radiation on the Development of Immunity to Heterologous Transplantation of Tumours.' Copenhagen (Levin & Munksgaard). —(1940) *Amer. J. Cancer*, **38**, 483.
Cornman, I.—(1951) *Science*, **133**, 37.
Gheorghiu, J.—(1926) *J. Path. Bact.*, **29**, 171.
Gordon, A. S. and Katsh, G. F.—(1949) *Ann. N.Y. Acad. Sci.*, **52**, 1.
Green, H. N. and Whiteley, H. J.—(1952) *Brit. med. J.*, ii, 538.
Greene, H. S. N.—(1938) *Science*, **88**, 357.—(1941) *J. exp. Med.*, **73**, 461.—(1947) *Cancer Res.*, **7**, 491.—(1948) *J. Amer. med. Ass.*, **137**, 1364.—(1950) *Yale J. Biol. Med.*, **22**, 611.—(1951) *Cancer Res.*, **11**, 529.
*Idem* and Lund, P. K.—(1944) *Ibid.*, **4**, 352.
Holden, M., Seegal, B. C. and Ryby, I.—(1951) *Amer. J. Path.*, **27**, 748.
Jensen, C. O.—(1902) *Hospitalstidende*, **10**, 489.—(1903) *Ibid.*, **11**, 549.
Kass, E. H., Kendrick, M. I. and Finland, M.—(1953) *Ann. N.Y. Acad. Sci.*, **56**, 737.
Keysser, F.—(1913) *Wien. klin. Wschr.*, **26**, 1664.
Kirschbaum, A.—(1951) *Cancer Res.*, **11**, 741.
Klein, G.—(1951) *Exp. Cell Res.*, **2**, 518.
Krebs, C. and Busch, F.—(1931) *Z. Krebsforsch.*, **34**, 234.
Loeb, L.—(1901) *J. med. Res.*, **6**, 28.
Murphy, J. B.—(1913) *J. exp. Med.*, **17**, 482.—(1914) *J. Amer. med. Ass.*, **62**, 1459.
Nicol, T. and Snell, R. S.—(1954) *Nature*, **174**, 554.
Patterson, W. B., Chute, R. N. and Sommers, S. C.—(1954) *Cancer Res.*, **14**, 656.
Pearson, O. H., Eliel, L. P. and Rawson, R. W.—(1950) *Proc. Clin. ACTH Conf.*, **1**, 318.
Ragan, C., Howes, E. L., Plotz, C. M., Meyer, K. and Blunt, J. W.—(1949) *Proc. Soc. exp. Biol., N.Y.*, **72**, 718.
Smirnova, E.—(1937) *Bull. Biol. Méd. exp. URSS*, **4**, 6.
Spain, D. M., Molomut, N. and Haber, A.—(1950) *Science*, **112**, 335.

SPIES, T. D., STONE, R. E., LOPEZ, G. G., MILLANES, F., TOCA, R. L. AND REBOREDO, A.—(1950) *Lancet*, ii, 241.
STOERK, H. C. AND SOLOTOROVSKY, M.—(1950) *Amer. J. Path.*, **26**, 708.
TAUBENHAUS, M.—(1953) *Ann. N.Y. Acad. Sci.*, **56**, 666.
TOOLAN, H. W.—(1951) *Proc. Soc. exp. Biol., N.Y.*, **77**, 572.—(1953) *Cancer Res.*, **13**, 389.—(1954) *Ibid.*, **14**, 660.
TOWBIN, A.—(1951*a*) *Ibid.*, **11**, 716.—(1951*b*) *Arch. Path.*, **52**, 199.
TYZZER, E. E.—(1916*a*) *J. Cancer Res.*, **1**, 117.—(1916*b*) *Ibid.*, **1**, 125.
WAGNER, A.—(1930) *Acta Radiol.*, **10**, 539.

### EXPLANATION OF PLATES

**FIG. 1.**—Metastatic skin tumour from human adenocarcinoma of oesophagus (Patient No. Z.1519). Haematoxylin-eosin. × 250.

**FIG. 2.**—Transplantation site in a mouse 45 days after subcutaneous grafting of human oesophageal adenocarcinoma shown in Fig. 1. Note preservation of structure. Haematoxylin-eosin. × 250.

**FIG. 3.**—Breast cancer of the patient No. R.64561. Adenocarcinoma with marked mucoid degeneration. Haematoxylin-eosin. × 65.

**FIG. 4.**—Solid carcinoma of cervix from patient No. R.64561. Haematoxylin-eosin. × 250.

**FIG. 5.**—Ascites smear from patient No. R.64561. Large number of tumour cells varying in size and shape. Pronounced admixture of granulocytes. Haematoxylin-eosin. × 365.

**FIG. 6.**—Three cortisone-treated mice and one control (left) 45 days after intra-abdominal inoculation of human ascites (from patient No. R.64561). Marked abdominal distention in all the treated mice.

**FIG. 7.**—Ascites smear from a cortisone-treated mouse 35 days after inoculation of the patient's ascites fluid. Tumour cells varying in size, shape and in number of nuclei. Pronounced vacuolation of the cytoplasm. Haematoxylin-eosin. × 425.

**FIG. 8.**—Solid tumour growth in a cortisone-treated mouse from the third passage of the ascites tumour H.A.1. Note existence of capillaries and the scarcity of stroma. Haematoxylin-eosin. × 300.

**FIG. 9.**—Tumour infiltration of kidney in an untreated mouse from the third passage of the ascites tumour H.A.1. Haematoxylin-eosin. × 300.

**FIG. 10.**—Tumour infiltration of pancreas in an untreated mouse from the fourth passage of the ascites tumour H.A.1. Haematoxylin-eosin. × 300.